# Implementation of Ultrasound-Guided Musculoskeletal Injections in a South Trinidad Medical Practice: A Perspective From a Resource-Limited Setting

**DOI:** 10.7759/cureus.101245

**Published:** 2026-01-10

**Authors:** Shiva Rampersad, Kevin Morris

**Affiliations:** 1 Department of Neurological Surgery, Evolve Medical, San Fernando, TTO; 2 Department of Research, Society for Brain Mapping and Therapeutics, Los Angeles, USA; 3 Department of Medical Education, Morris Lifesciences Center for Innovation and Research, Nagpur, IND; 4 Department of Research, Aklun Biotech LLC, Nagpur, IND

**Keywords:** hyaluronic acid, interventional ultrasonography, intra-articular injections, knee osteoarthritis, low- and middle-income country, musculoskeletal pain, platelet-rich plasma, point-of-care systems, resource-limited settings, tendinopathy

## Abstract

Musculoskeletal pain (MSP) is a general term for pain varying by source, characterized into nociceptive (tissue pain), neuropathic (nerve pain), and nociplastic (centralized pain). It affects muscles, bones, joints, tendons, and ligaments due to multiple etiologies, including trauma, degenerative changes, or autoimmune and inflammatory disorders, presenting as either acute or chronic pain with symptoms such as sharp, dull, tingling, or burning sensations. It is a leading cause of disability globally, particularly in low and middle-income countries. We want to highlight a real-world implementation that aims to illustrate translational feasibility, and not a comparative efficacy trial on degenerative (wear and tear) conditions such as osteoarthritis and tendinous injuries. Ultrasound-guided musculoskeletal (MSK) injections offer a minimally invasive and accurate treatment option for these two conditions, especially in settings where patients can face barriers to surgical care. Traditionally, management of such cases includes analgesia, physiotherapy, and surgery. This article highlights the implementation of ultrasound-guided musculoskeletal injection procedures in a general medical and surgical clinical setting in southern Trinidad, including hyaluronic acid (HA), platelet-rich plasma (PRP), and diluted corticosteroid injections. Over the course of two years (2023-2025), a total of 115 adult patients with chronic MSP who had an unsatisfactory response to treatment with oral analgesics were treated under ultrasound guidance. The patients largely reported qualitative improvement in pain and increased functionality post-intervention. No major complications due to the procedures were observed. Through this perspective/observational experience, we do not aim to quantify the treatment effect sizes, but rather to bring to attention the practical procedural techniques, associated safety, potential workflow advantages, and practical considerations of ultrasound-guided musculoskeletal injections, thereby highlighting their use as a radiation-free, feasible, and efficient intermediate treatment option in resource-limited settings that have limited access to advanced imaging or surgical services.

## Editorial

Globally, musculoskeletal disorders (MSKD) and musculoskeletal pain (MSP) affect millions of people and are known to contribute to functional decline, disability, and reduced quality of life [1,2]. MSP affects parts of the musculoskeletal system, such as the muscles, ligaments, tendons, bones, or joints, due to various factors such as trauma, autoimmune/inflammatory disorders, and degenerative diseases, and can present as either acute or chronic pain, characterized by source into nociceptive (tissue pain) [3,4], neuropathic (nerve pain) [5,6], and nociplastic (centralized pain) [7,8]. Conventionally, the management of patients with MSP includes analgesics, physiotherapy, and surgery.

In the Caribbean countries, including the Republic of Trinidad and Tobago, the management of MSKD and similar degenerative joint diseases and tendinopathies is either conservative with oral medications such as nonsteroidal anti-inflammatory drugs (NSAIDs) and opioids coupled with physiotherapy, or a surgical approach, which often involves pre-procedure imaging studies such as fluoroscopy or magnetic resonance imaging (MRI). The conservative route is often the go-to option as it is non-invasive and more affordable to most patients. A surgical option is often very expensive, given the fact that most patients lack medical insurance coverage, coupled with the prolonged waiting time for orthopedic surgeries (1,610 patients currently awaiting a surgical slot) in the public health sector hospitals of Trinidad, as per a recent report from 2025 [9]. A non-surgical but potentially cost-effective and resource-efficient intermediate approach is needed to reduce imaging costs and eliminate opioid dependence and medication-related complications such as NSAID-induced gastritis, peptic ulcers, and persistent pain-induced depression. Point-of-care ultrasound (POCUS) guided treatment has emerged as a portable, affordable, and effective tool for diagnosis and a real-time navigational image-based procedure to ensure accurate placement of the product, particularly in resource-limited settings in low- and middle-income countries [10,11]. It has helped clinicians ensure accurate needle placement and minimize complications. We describe how we used ultrasound-guided musculoskeletal (MSK) injections, including hyaluronic acid (HA), platelet-rich plasma (PRP), and diluted corticosteroid formulations to fill the critical gap between conventional and surgical management in a resource-limited private outpatient clinic in South Trinidad.

Setting and patient population

A total of 115 adult patients, aged 18-75 years, from the Republic of Trinidad and Tobago were followed up in a private general medical-surgical clinic in the city of San Fernando over two years from 2023 to 2025. These patients had complaints of joint and/or tendinous pain involving the knee, lumbar spine, or shoulder, all having pain that persisted for more than 12 weeks despite oral analgesics, which was considered significant enough to affect their quality of life. All procedures were performed by a licensed medical doctor who has training in neurological surgery, spine surgery, sports medicine, and pain management. Informed verbal consent was obtained prior to any intervention or procedure. Table 1 provides patient demographics and clinical characteristics.

**Table 1:** Patient demographics and clinical characteristics NSAIDs: nonsteroidal anti-inflammatory drugs

Variable	Data comment
Age range	18-75 years
Sex distribution	Approximate equal male to female distribution
Primary diagnoses	Osteoarthritis (knee/shoulder), patellar tendinopathy, rotator cuff tendinopathy, lumbar spondylosis, lumbar radiculopathy
Duration of symptoms	More than 12 weeks for all patients
Prior treatments	Oral NSAIDs, opioids, and physiotherapy

Equipment

The clinic uses United States Food and Drug Administration (US-FDA)-cleared portable wireless ultrasound machines equipped with a high-frequency linear scanner, model L15 HD_3_ (5-15 MHz) (Clarius Mobile Health, Vancouver, BC, Canada), for superficial structures up to 7 cm depth, and a convex scanner, model C3 HD_3_ (2-6 MHz) (Clarius Mobile Health, Vancouver, BC, Canada), for deeper lumbar anatomy up to 40 cm depth [12]. The linear scanner is useful for patients weighing less than 70-80 kg having a body mass index (BMI) less than or equal to 24.9, and the convex scanner is useful for obese patients with a BMI over 25.

Technique and general workflow

**Figure 1:**
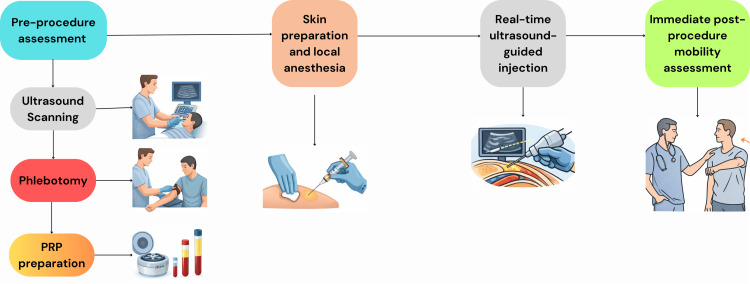
Illustration of the technique and general workflow PRP: platelet-rich plasma Image created using Adobe Illustrator and Canva

The general workflow and techniques used at the clinic are visualized in a flowchart in Figure 1.

We want to present some real case images below for a clearer understanding and better visualization. Informed verbal consent was obtained from the patients for the publication of the accompanying images.

**Figure 2:**
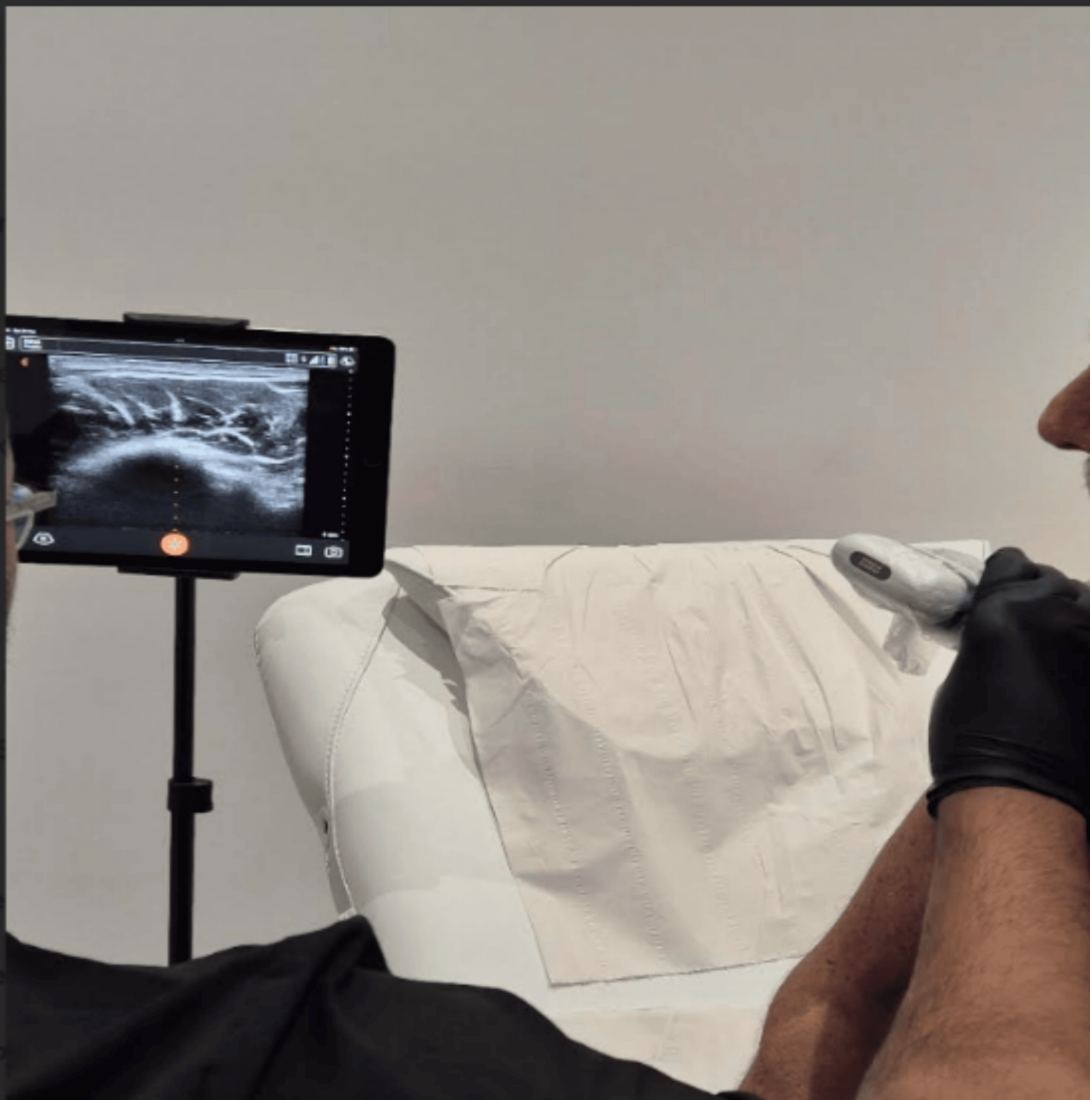
Procedure of initial ultrasound scanning

**Figure 3:**
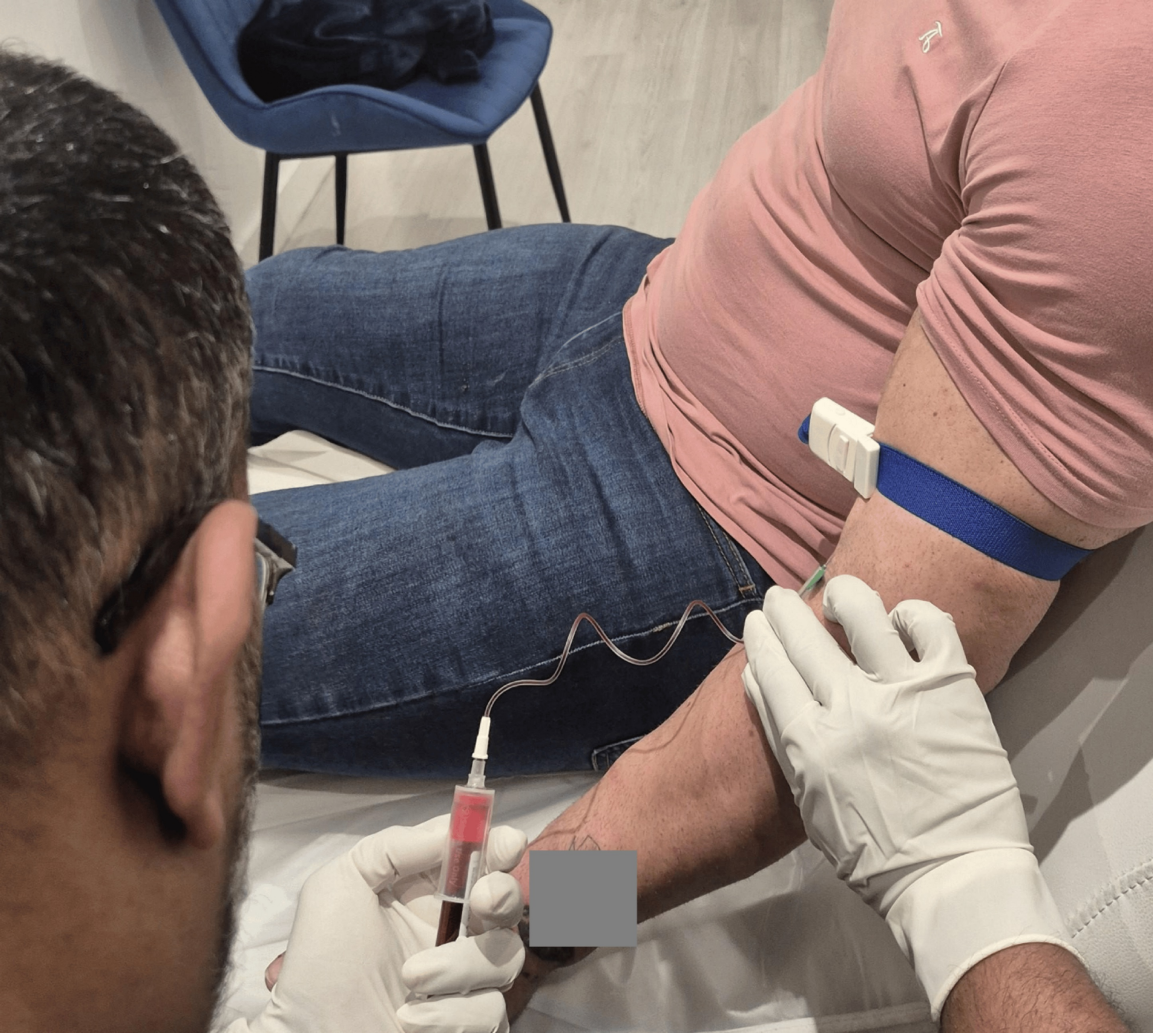
Procedure of phlebotomy

**Figure 4:**
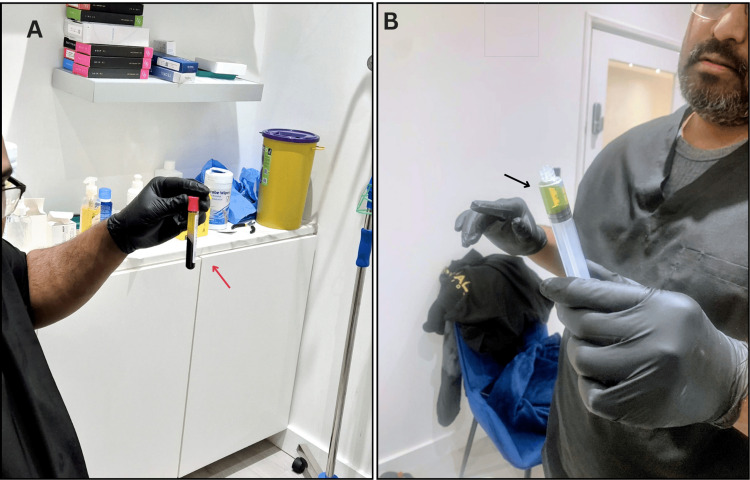
Preparation of plasma-rich platelet: (A) post-centrifuge blood sample showing PRP in the collection tube (red arrow) and (B) prepared yellow PRP ready for use (black arrow) PRP: platelet-rich plasma

The pre-procedure assessment of the patient includes the initial ultrasound scanning (Figure 2), phlebotomy (Figure 3), and preparation of platelet-rich plasma (Figure 4A and Figure 4B).

**Figure 5:**
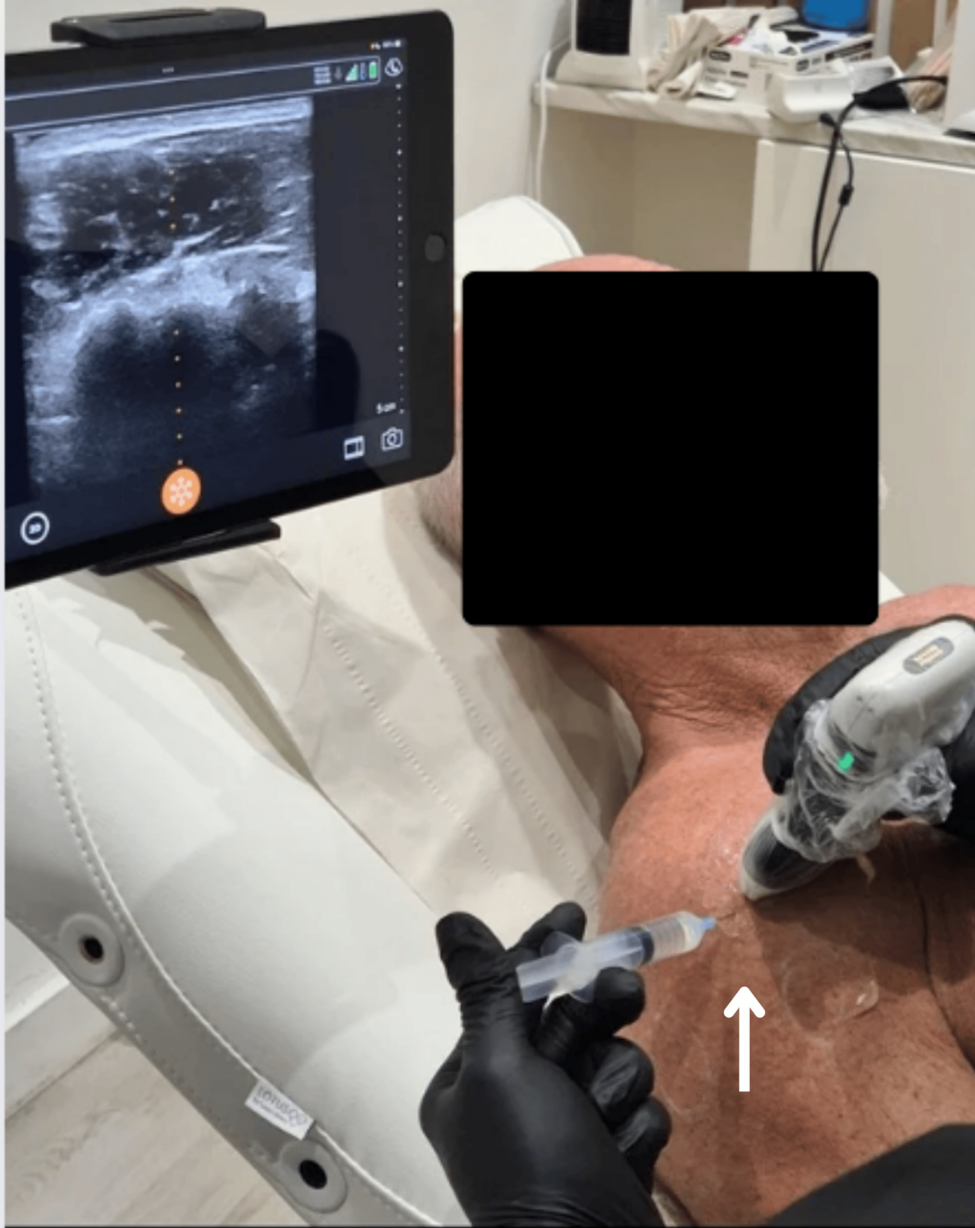
Administration of local anesthesia under ultrasound guidance (white arrow) post skin preparation

Administration of local anesthesia under ultrasound guidance post skin preparation is shown in Figure 5.

**Figure 6:**
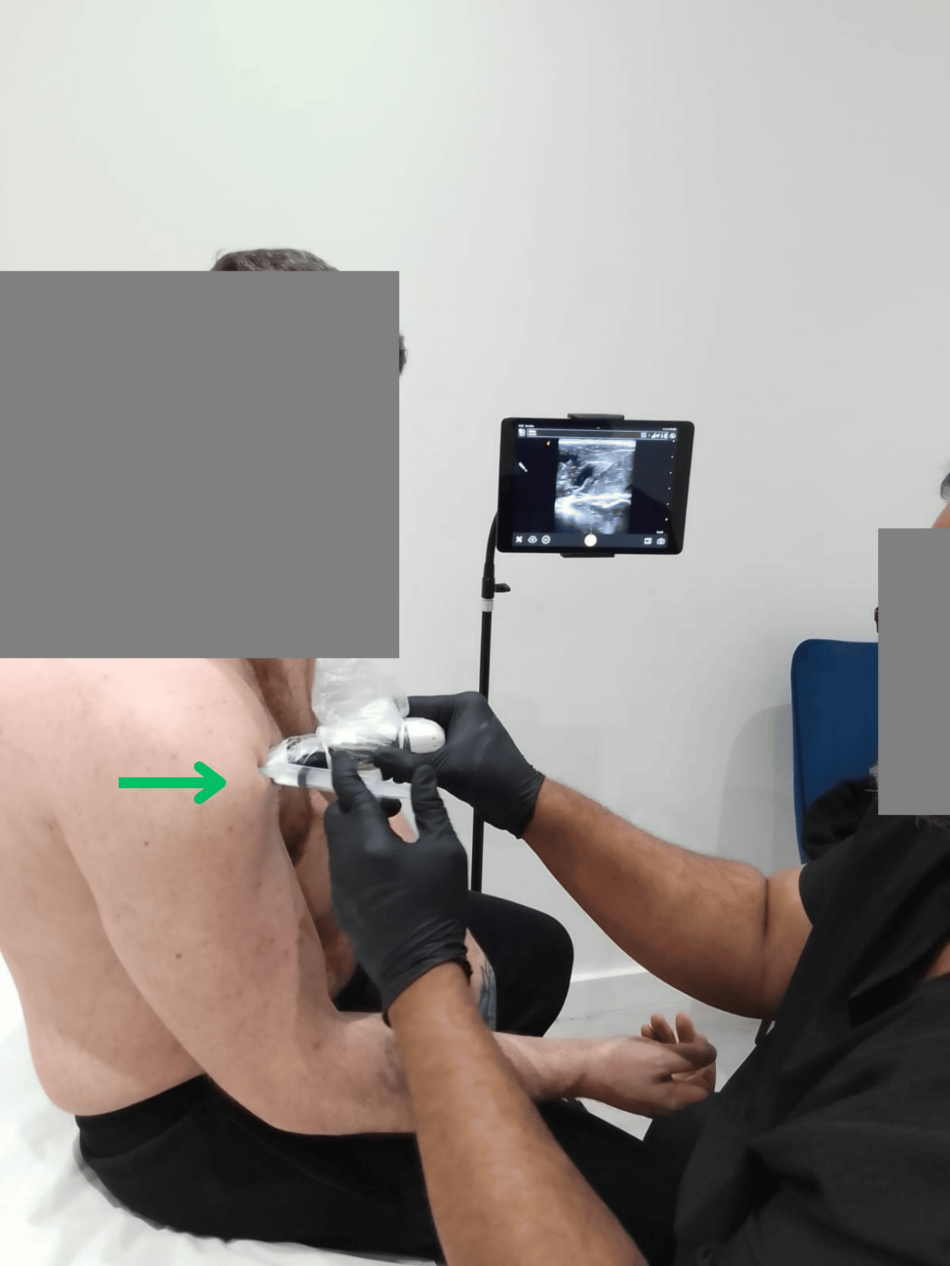
Real-time ultrasound-guided injection delivery of injectate (green arrow)

Administration of injectate, such as PRP/HA/corticosteroids, in real-time under ultrasound guidance is presented in Figure 6.

**Figure 7:**
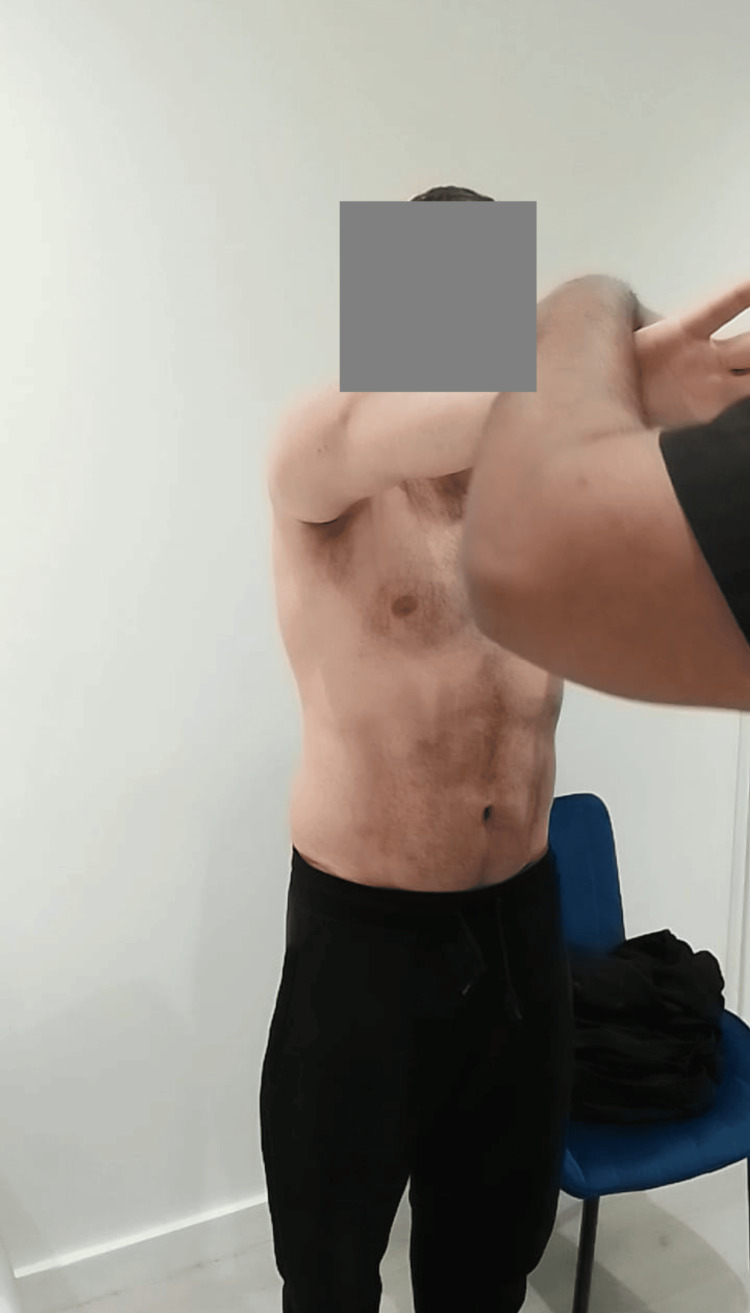
Post-procedural functional mobility evaluation

Post-procedural functional mobility evaluation is shown in Figure 7.

Interventions performed

The patient allocation to either PRP, HA, or diluted corticosteroid was based solely on practical clinical decision-making, patient-specific factors such as the underlying pathology, presenting symptoms, prior response to conservative treatments, and imaging findings where available, and no randomization or comparative intent was intended. The three intervention groups were meant to reflect individualized, indication-driven treatment approaches rather than a head-to-head comparison. A total of three minimally invasive treatment options were implemented as described below.

Platelet-Rich Plasma Injection (Number of Patients in the Cohort: n = 57)

Blood is drawn from the patient and then centrifuged to obtain the yellow PRP (an average of 4-5 mL per area is advised) [13]. In this cohort of patients, the yellow PRP is then injected under ultrasound guidance into the affected joint (such as the knee, shoulder, or facet joints) or affected tendon (such as the patellar or rotator cuff tendons). Patients typically returned to work the same day, thus proving the effectiveness of treatment and the negligible recovery time. Qualitative reports highlight a meaningful reduction in pain and improved function, usually beginning 4-6 weeks post-injection. PRP contains stem cells and growth factors that slowly begin to work with results lasting for up to 6-8 months [14].

Hyaluronic Acid Injection (Number of Patients in the Cohort: n = 16)

HA fillers approved by the US-FDA for degenerative conditions such as osteoarthritis of the knee joint provide a gel-like cushion for worn-out articular surfaces [15]. In this cohort of patients with moderate to severe osteoarthritis, who had a history of daily use of NSAIDs and opioids, with X-ray features of disease that qualify patients for expensive surgical intervention, HA was injected into the knee joint with the suprapatellar recess approach. Patients reported qualitative symptomatic improvement lasting for 8-12 months. This is consistent with evidence that HA provides a gel-like mechanical cushioning for such degenerative articular surfaces [15].

Diluted Corticosteroid Injection (Number of Patients in the Cohort: n = 42)

A diluted corticosteroid injection can also be used either as a single treatment or combined with either PRP or HA treatment modalities [16]. For this cohort of patients, a mixture containing depomedrol (40-80 mg/dL), 2% lidocaine (2 mL), and bacteriostatic saline (5 mL) was injected at intervals of 4-6 months into the affected joints to reduce long-term complications of steroids such as avascular necrosis, gastrointestinal ulcers, and metabolic disturbances. This modality can be used on all joints. However, with tendon injuries, use was limited to a “one-off use” in severe pain due to the long-term risk of iatrogenic tendon rupture [17].

Table 2 gives an overview of the distribution of the interventions that were performed along with the anatomical sites.

**Table 2:** Distribution of interventions performed Patient distribution reflects indication-based clinical decision-making rather than comparative group assignment.

Intervention type	Number of patients	Anatomical sites
Platelet-rich plasma injection	57	Knee, shoulder, patellar tendon, rotator cuff, facet joints
Hyaluronic acid injection	16	Knee joint
Diluted corticosteroid injection	42	Knee, shoulder, lumbar spine, other joints

Ultrasound-guided approaches

Knee Joint Region (Suprapatellar Recess Technique)

**Figure 8:**
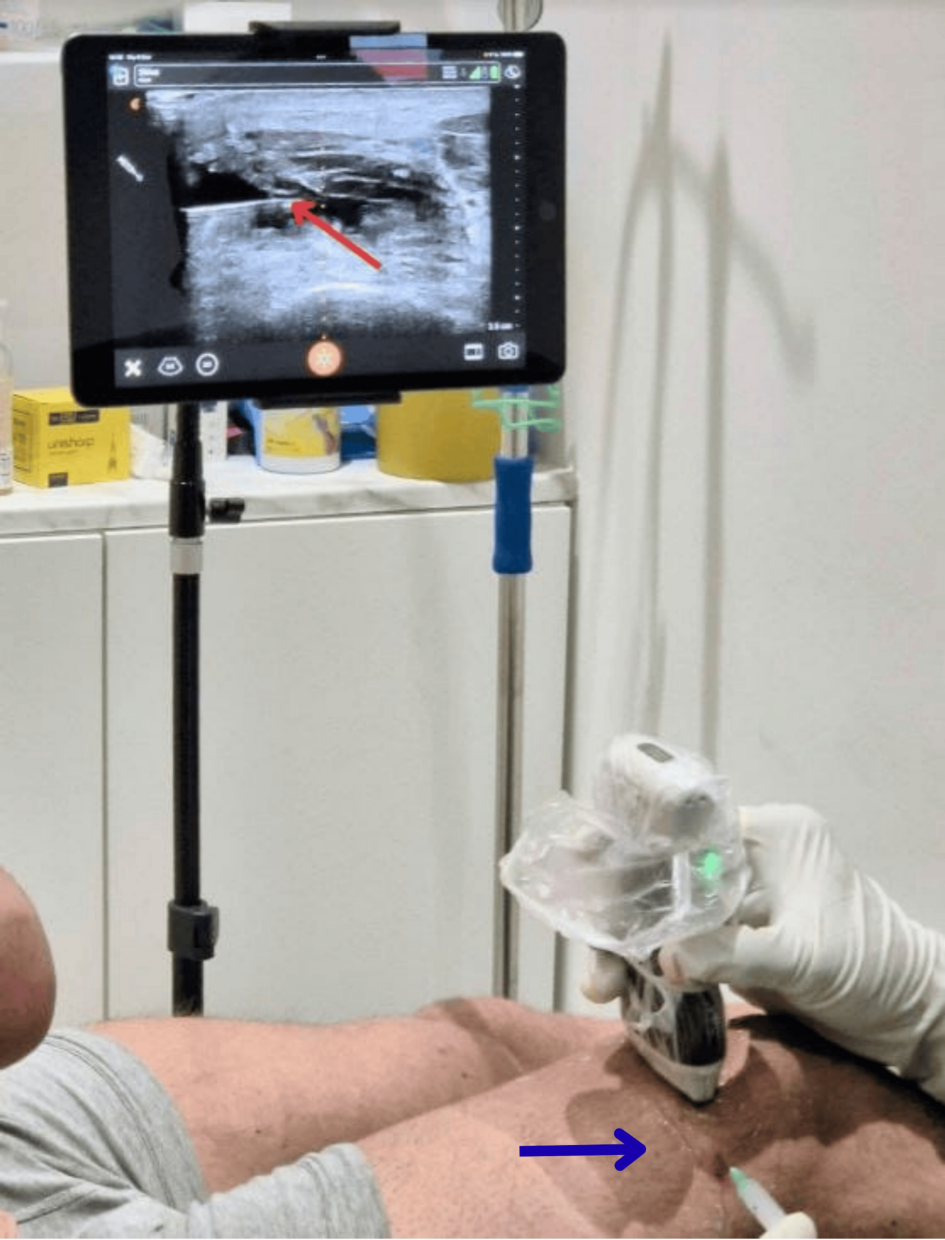
Suprapatellar recess technique (lateral-to-medial in-plane approach) for medication delivery by injection under ultrasound guidance (blue arrow) in the knee joint The needle well can be seen on the monitor screen as it enters the suprapatellar bursa (red arrow).

Superficial injections of anatomical structures such as the Achilles tendon are technically easier without the need for image guidance. However, deeper injections involving the knee joint and the shoulder joint (rotator cuff) can be challenging. Traditionally, knee joint injections are done by entering through the anterior joint line. In this approach, there is a greater risk of injuring the articular surface, which is a major pain generator, and there is added risk of causing injury to the vascular structures [18]. With the help of the POCUS device, we adopted a suprapatellar recess technique, as demonstrated in Figure 8, which guides our needle trajectory from a lateral-to-medial in-plane approach under direct ultrasound vision [19]. This significantly minimized the risk of contacting articular cartilage and improved accuracy.

Shoulder Region

**Figure 9:**
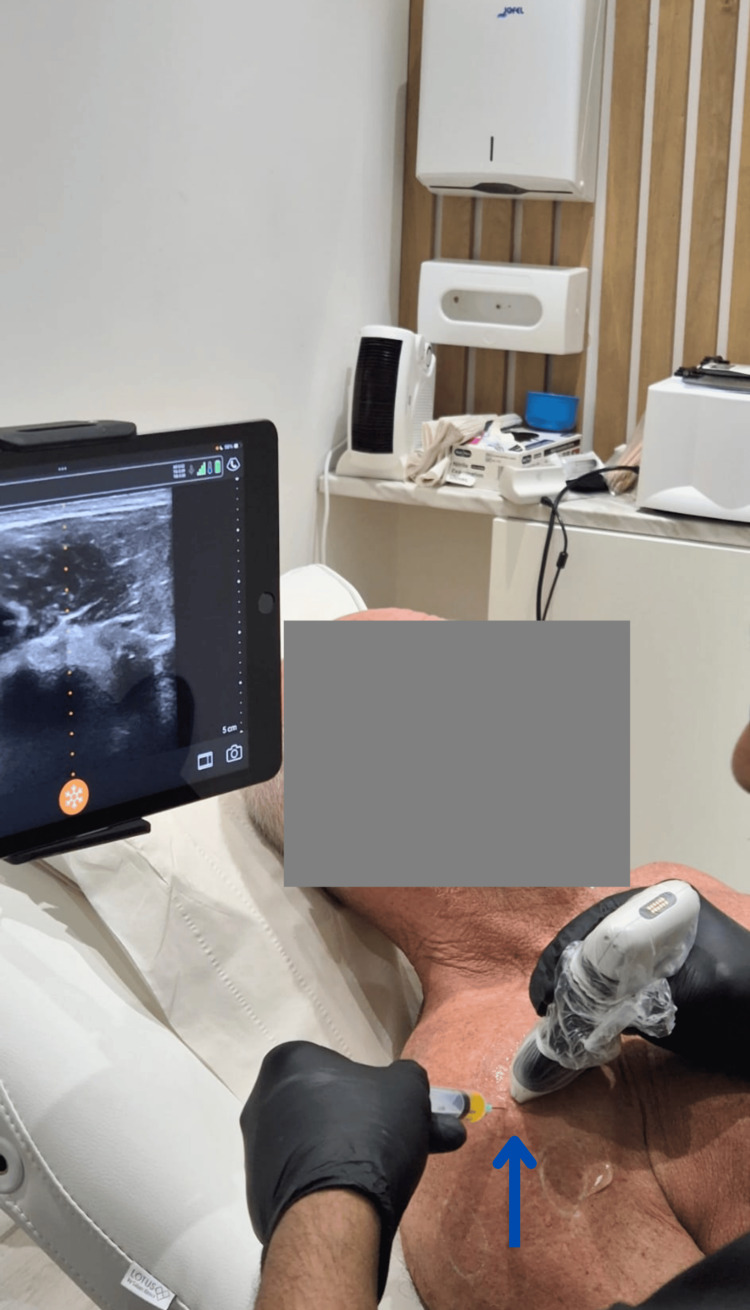
Anterior subacromial approach for injection of yellow PRP under ultrasound guidance (blue arrow) into the proximal long head of the biceps tendon PRP: platelet-rich plasma

With the aid of a POCUS device, a variety of pathologies can be visualized, including, but not limited to, adhesive capsulitis (frozen shoulder), impingement pathology, acromioclavicular joint disease, rotator cuff tendinopathies and intratendinous tears (partial versus complete), glenohumeral joint osteoarthritis, and proximal long head of biceps tendon injuries. There is the added advantage of dynamic assessment of the involved musculotendinous complex with active/passive movement of the joint. Ultrasonography assessment also allows measurement of the shortest and safest trajectory for needle placement, allowing the clinician real-time confirmation of accurate drug delivery [18]. In Figure 9, we demonstrate the anterior subacromial approach for injection of a drug (in this case, diluted corticosteroids containing lidocaine 2% and 40 mg/mL depomedroxyprogesterone) under ultrasound guidance into the proximal long head of the biceps tendon.

Lumbar Spine Region (Facet and Transforaminal Epidural Injection)

**Figure 10:**
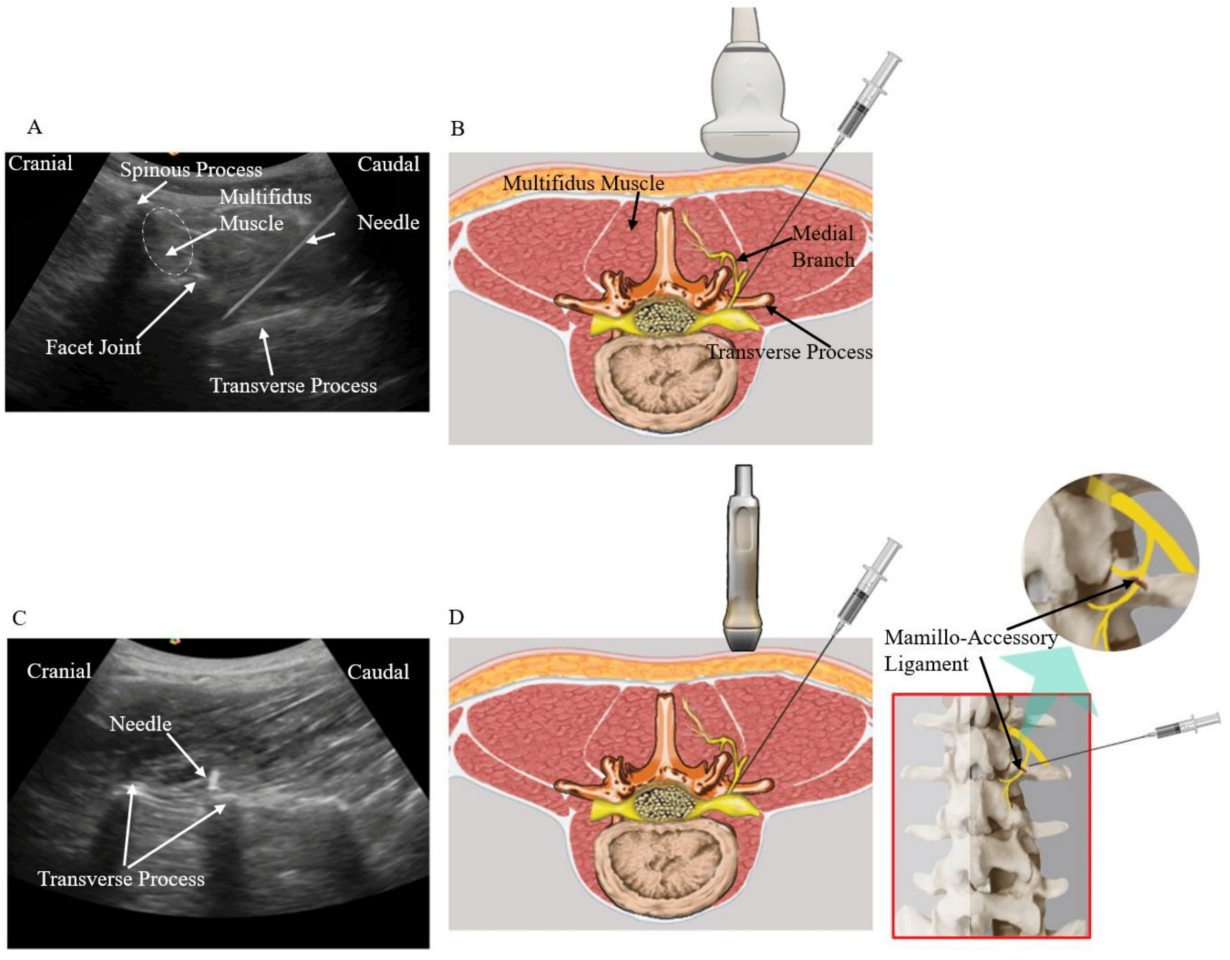
Depiction of a typical ultrasound-guided facet joint steroid injection (A) Sonographic cross-axis view of the lumbar region. (B) Depiction of a low-frequency convex transducer, facet joint, multifidus muscle, and transverse process. Needle inserted at an angle of 45° in a lateral to medial direction and navigated to the junction between the superior articular process and the superior border of the transverse process. (C and D) The transducer is subsequently rotated 90° to obtain a longitudinal view of the transverse process to ensure the tip of the needle is located at the cranial edge of the transverse process (hyperechoic spot). (D) The location of the mamillo-accessory ligament is shown for reference. © Suputtitada et al. (2023) [21] Image used under Creative Commons Attribution (CC BY) license (https://creativecommons.org/licenses/by/4.0/)

Generally, a radiographer is needed to operate a fluoroscopic C-arm when treating mechanical back pain with traditional fluoroscopic facet joint injections and transforaminal lumbar epidurals for radiculopathy. This requires wearing protective lead jackets and neck pieces against ionizing radiation, which increases the need for more trained staff on site and prolongs the overall time of treatment. With proper patient selection and the use of POCUS, a trained physician can perform a single-level facet and transforaminal epidural injection in 10-15 minutes (from the time of laying the patient in supine or lateral decubitus position to completion of treatment) while eliminating radiation exposure. Such efficiency is useful in a busy outpatient clinical setting with minimal costs while providing similar efficacy to conventional fluoroscopy [20]. In Figure 10, as has been depicted by Suputtitada et al. (2023) [21], we can see that the ultrasound-guided lumbar facet joint injections start with a cross-axis scan of the lumbar region using the appropriate transducer, and key anatomical landmarks such as the facet joint, multifidus muscle, and transverse process are identified. Introduction of the needle is done at an angle of 45° in a lateral-to-medial direction toward the junction of the superior articular process and the superior border of the transverse process. The transducer is then rotated 90° to acquire a longitudinal view of the transverse process and confirm the precise placement of the needle tip at the cranial edge, which is seen as a hyperechoic focus. The mamillo-accesory ligament serves as an additional anatomical landmark [21].

Discussion

**Figure 11:**
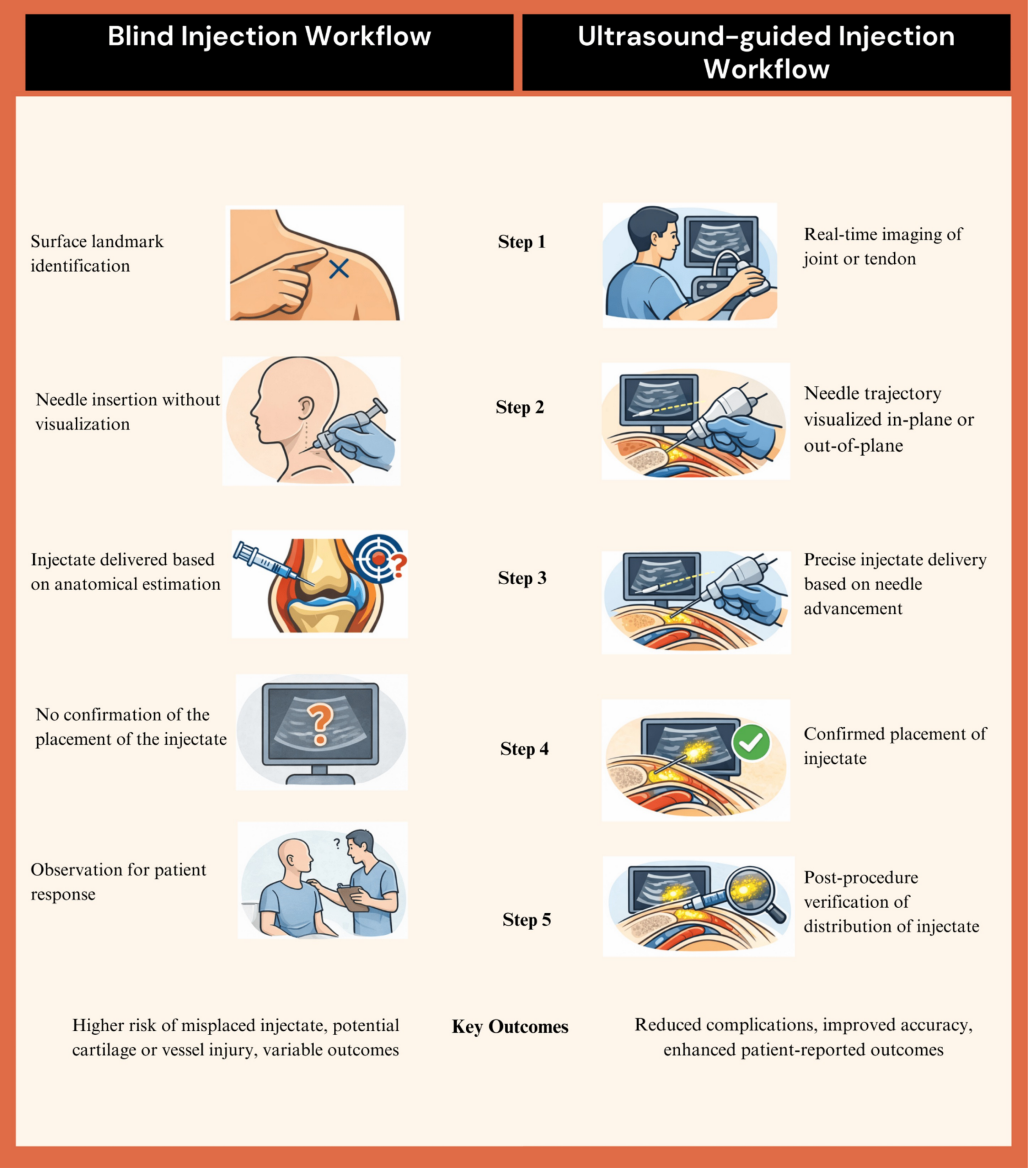
Workflow comparison: blind versus ultrasound-guided injections Image created using Adobe Illustrator and Canva

The primary objective of this perspective article is to bring forward the potential clinical and systemic benefits of POCUS injections that are above traditional conservative management and also landmark-based injection approaches. Ultrasound guidance has been known to improve procedural accuracy and overall safety [22], which in turn provides added benefits to the patients in the form of reduced costs, reduced dependence on drugs, and faster access to treatment. It has been a traditional practice in Trinidad for MSP treatments to be performed “blindly,” utilizing anatomical surface landmarks without the use of image guidance. This is purely down to the lack of availability of resources and training in the use of such technology. POCUS treatment provides real-life dynamic radiological assessment of the affected area, providing further diagnostic information beyond prior standard X-rays. While the use of computed tomography (CT) and MRI provides better anatomical delineation and soft tissue assessment, respectively, these are more expensive, are not readily available or accessible in resource-limited settings, provide static rather than dynamic imaging, and have no practical role in allowing concurrent treatment in primary care. It is important to understand the differences in treatment strategies and outcomes between blind and ultrasound-guided injection workflows, as illustrated through a five-step protocol in Figure 11, which can help clinicians globally in improving the quality of care for patients.

The patient outcomes were assessed qualitatively during follow-up visits. In Table 3, we highlight the outcome domains, associated observed contextual findings, and their relevance from a healthcare point of view. The outcomes are based purely on qualitative clinical follow-up and observational assessments. The findings are intended to understand the implementational feasibility, safety, and holistic value. It is also important to note that tendon injections must be given cautiously to avoid the risk of tendon rupture.

**Table 3:** Clinical and system-level outcomes observed following ultrasound-guided musculoskeletal injections MSK: musculoskeletal, NSAIDs: nonsteroidal anti-inflammatory drugs, MRI: magnetic resonance imaging

Outcome domain	Observed findings	Interpretation/clinical relevance
Pain response	The majority of patients reported subjective reduction in pain intensity during follow-up visits.	This suggests symptomatic benefit consistent with published evidence on ultrasound-guided MSK injections; it was not measured quantitatively.
Functional status	Patients commonly reported improved tolerance for daily activities and mobility.	It indicates functional relevance beyond pain relief, particularly important in working-age population groups.
Return to activity or work	Most patients were able to return to routine activities or work on the same day as the procedure.	This reflects minimal procedural downtime and feasibility in busy outpatient settings.
Medication dependence	Patients reported a reduction in reliance on NSAIDs and opioids following intervention.	This highlights the potential opioid- and NSAID-sparing role of image-guided interventional pain management.
Procedural safety	No major procedural complications were observed during the study period.	This supports the safety profile of ultrasound-guided injections when performed by trained clinicians.
Procedure efficiency	Procedures are typically completed within a short outpatient visit without the need for additional imaging staff.	It demonstrates workflow efficiency compared with MRI or fluoroscopy-based interventions.
Imaging and radiation exposure	No exposure to ionizing radiation needed.	Relevant advantage in settings lacking fluoroscopy and for reducing cumulative radiation exposure.
Access to care	Enabled timely intervention in patients facing prolonged waits for orthopedic surgery.	This demonstrates the role as an intermediate therapeutic option between conservative care and surgery.
Feasibility in a resource-limited setting	Successfully implemented in a small private outpatient clinic with portable ultrasound equipment.	This demonstrates scalability and adaptability in low- and middle-income healthcare settings.
Cost and logistical considerations	Elimination of fluoroscopy equipment, dedicated radiology staff, and radiation safety requirements; reduced downstream medication use; facilitated access to care for uninsured patients and those facing prolonged surgical waitlists.	This highlights it to be a resource-efficient, accessible intermediate therapeutic option in constrained health systems.

We want to highlight that POCUS MSK injections can be very effective and serve as a viable intermediate therapeutic option that perfectly fills the gap between conservative care and surgical management when indicated in the outpatient settings of Caribbean or other low- and middle- income countries where there are financial and other constraints, such as a lack of infrastructure that can limit access to surgical treatment or fluoroscopic guidance. In this article, we do not aim to quantify treatment effect sizes or state any superiority of injectables or treatment protocols used in the clinic.

Limitations

Although there are several benefits to the methods and approaches described in the article, we would like to point out some limitations. The data is representative of observations at a single clinical site. It can be challenging to replicate similar positive results at other clinical sites without proper training in the use of ultrasonography technology, neurology, neurosurgery, or pain management. The outcomes were not measured using standardized pain scales or functional scores, and no formal cost-effectiveness analyses were conducted. The use of the specific Clarius portable ultrasound scanners is not a limitation in itself, but portable handheld ultrasound scanners from other companies without advanced software that is available in the Clarius models can possibly act as a hindrance or limitation in some clinical settings.

Conclusions

With the global rise of musculoskeletal disabilities, prolonged surgical wait times in low- and middle-income countries, and the ever-increasing threat of opioid dependence, the experience we have gained through our work will help other clinicians in helping their patients. The importance of this perspective is not in showing the superiority of any particular injectate but in demonstrating a radiation-free, scalable, and workflow-efficient model that can easily be replicated or adopted. In well-funded public healthcare settings, the methods we adopted in our single clinic will work wonders and help reduce the long wait times for patients in receiving quality care. Opioid dependence can be cut down, and the opioid crisis can be averted. Ultrasound-guided MSK injections provide a safe, accurate, and, in our opinion, potentially cost-saving and resource-efficient intermediate treatment option for patients with chronic joint and soft tissue pain in resource-limited settings. Future prospective studies that incorporate standardized pain scores, have longer follow-up, and do economic benefit analyses are warranted. While not a replacement for surgical intervention when indicated, these procedures serve as an effective adjunct in treatment that reduces medication dependence and improves quality of life.
